# Neurally adjusted ventilatory assist decreases work of breathing during non-invasive ventilation in infants with severe bronchiolitis

**DOI:** 10.1186/s13054-019-2379-8

**Published:** 2019-04-16

**Authors:** Florent Baudin, Guillaume Emeriaud, Sandrine Essouri, Jennifer Beck, Etienne Javouhey, Claude Guerin

**Affiliations:** 1 Hospices Civils de Lyon, Hôpital Femme Mère Enfant, Réanimation Pédiatrique, 59 Bd Pinel, F-69500 Bron, France; 2 University Lyon, Université Claude Bernard Lyon1, Ifsttar, UMRESTTE, UMR T_9405, F-69373 Lyon, France; 3 Pediatric Intensive Care Unit, CHU Sainte-Justine, Université de Montréal, Montreal, QC Canada; 4 Department of Pediatrics, CHU Sainte-Justine, Université de Montréal, Montreal, QC Canada; 5 Keenan Research Centre for Biomedical Science, Li Ka Shing Knowledge Institute, St. Michael’s Hospital, Toronto, ON Canada; 6 Institute for Biomedical Engineering and Science Technology (iBEST), Ryerson University and St-Michael’s Hospital, Ontario, Canada; 7 Department of Pediatrics, University of Toronto, Toronto, Ontario Canada; 8 Hospices Civils de Lyon, Hôpital de la Croix-Rousse, Médecine Intensive Réanimation, F-69004 Lyon, France; 9 Université de Lyon, Université Claude Bernard Lyon 1, Villeurbanne, France; 10 INSERM 955 – Eq13, Institut Mondor de Recherche Biomédicale, Créteil, France; 11 Médecine Intensive-Réanimation, Grenoble, France; 12 INSERM 1042 HP2, Grenoble, France

Dear Editor,

Though neurally adjusted ventilatory assist (NAVA) is known to improve patient-ventilator interactions in infants with bronchiolitis [1, 2], its impact on respiratory muscles unloading has not previously been studied.

We conducted a secondary analysis (ethics committee approval CE_SRLF_18-48) of a prospective physiological study [3] which evaluated the impact of body positioning on work of breathing (WOB) in infants with severe bronchiolitis. Seven of the children included (median age 35 [27–63] days) had a respiratory recording during the transition from nasal continuous positive airway pressure (nCPAP; set at 7cmH_2_O [4]) to NAVA. Esophageal (Peso), gastric (Pga), and airway (Paw) pressures, as well as Electrical activity of the diaphragm (Edi), and flow were recorded simultaneously. Median NAVA level was set at 0.7 [0.7–0.9] cmH_2_O/μV and median positive end expiratory pressure at 5 [4–7] cmH_2_O. Twenty-five breaths during the last 2 min in nCPAP then during the first 2 min in NAVA were analyzed off-line. Metabolic cost of breathing was estimated by the Peso (PTPeso) and diaphragmatic (PTPdi) pressure time product, inspiratory effort by the Peso (ΔPeso) and diaphragmatic (ΔPdi) pressure swings, and respiratory drive by the Edi swing (ΔEdi). Data were expressed as median [IQR] and compared using Wilcoxon two-sample paired sign test. A *p* value < 0.05 was considered significant.

As detailed in Table 1 and illustrated in Fig. 1, all indices of WOB (PTPeso, PTPdi, ΔPeso, ΔPdi, Edi swing, and inspiratory time to total time ratio (Ti/Ttot)) decreased significantly in every child with NAVA as compared to nCPAP (*p* < 0.05 in all instances), while the mean Paw was increased (*p* < 0.05).

**Table 1:** Comparison of physiological parameters between nasal continuous positive airway pressure and neutrally adjusted ventilatory assist

	nCPAP	NAVA	p*
Ti/Ttot (%)	0.47 [0.45–0.49]	0.40 [0.37–0.45]	0.02
Respiratory rate (/min)	71 [64–84]	65 [57–80]	0.31
Mean airway pressure (cmH_2_O)	7.0 [6.9–7.1]	10.6 [9.4–11.9]	0.02
ΔEdi (μV)	19 [17–25]	16 [10–19]	0.03
Swing Peso (cmH_2_O)	14 [12–18]	8 [8–13]	0.01
Swing Pdi (cmH_2_O)	14 [13–15]	10 [9–10]	0.02
PTPeso/breath (cmH_2_O s)	4.7 [3.4–6.1]	2.1 [1.9–3.7]	0.02
PTPdi/breath (cmH_2_O s)	4.2 [3.9–4.4]	2.6 [2.5–2.8]	0.02
PTPeso/min (cmH_2_O s/min)	365 [237–429]	162 [139–226]	0.02
PTPdi/min (cmH_2_O s/min)	298 [256–354]	157 [151–199]	0.02

Data are expressed as median [interquartile range]

*nCPAP* nasal continuous positive airway pressure, *NAVA* neutrally adjusted ventilatory assist, *PEEP* positive end expiratory pressure, *Ti* inspiratory time, *Ttot* total time, *Peso* esophageal pressure, *Pga* gastric pressure, *Edi* electrical activity of the diaphragm, *PTP* pressure time product

*Wilcoxon two-sample paired sign test

**Fig. 1:**
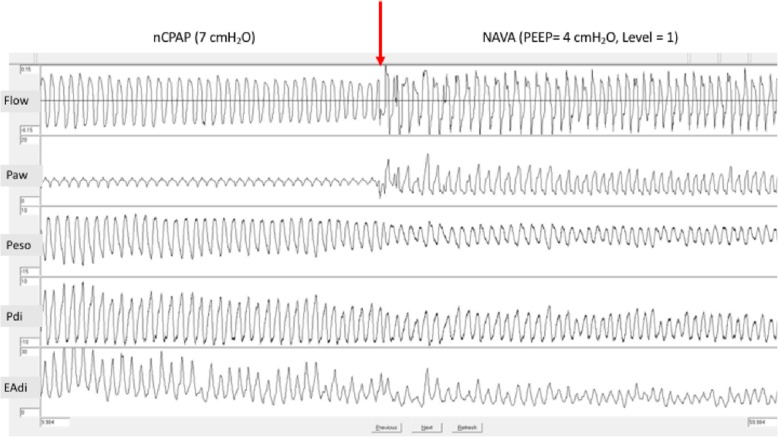
Decrease of esophageal and trans-diaphragmatic pressure swing and Edi amplitude after switching to neurally adjusted ventilatory assist. The red arrow indicates the switch from nCPAP to NAVA. nCPAP, nasal continuous positive airway pressure; NAVA, neurally adjusted ventilatory assist; PEEP, positive end expiratory pressure; Paw, airway pressure; Peso, esophageal pressure; Pga, gastric pressure; EAdi, electrical activity of the diaphragm

In this physiological study, we report an improvement of respiratory unloading by adding a second level of pressure with NAVA in infants with severe bronchiolitis. WOB decreased immediately after switching to NAVA (Fig. 1), as reported previously in adults with obstructive lung diseases [5], and was associated with a lower neural drive and Ti/Ttot ratio.

This study has several limitations, including the small sample size, the short study period, the non-randomized order of recordings, and the non-standardized NAVA settings. However, the consistent, rapid, and large improvement in WOB-related indices observed in every infant is an important finding, especially considering the number of infants with severe bronchiolitis who may benefit from an improvement in non-invasive support. The findings support the need for further evaluation of the potential interest of NAVA to improve the efficiency of non-invasive support in infants with bronchiolitis.

## Abbreviations

Edi: Electrical activity of the diaphragm
IQR: Interquartile range
NAVA: Neurally adjusted ventilatory assist
nCPAP: Nasal continuous positive airway pressure
Paw: Airway pressure
Peso: Esophageal pressure
Pga: Gastric pressure
PTP: Pressure time product
WOB: Work of breathing
